# The Proangiogenic Potential of Rat Adipose-Derived Stromal Cells with and without Cell-Sheet Induction: A Comparative Study

**DOI:** 10.1155/2022/2601764

**Published:** 2022-10-05

**Authors:** Xiaoru Xu, Shuang Song, Xiangdong Liu, Yuchao Zhou, Shaojie Shi, Guoqiang Zhao, Xingxing Wang, Xutao Chen, Wenshuang Zhao, Sijia Zhang, Wei Ma, Yingliang Song

**Affiliations:** ^1^State Key Laboratory of Military Stomatology & National Clinical Research Center for Oral Diseases & Shaanxi Engineering Research Center for Dental Materials and Advanced Manufacture, Department of Implantology, School of Stomatology, The Fourth Military Medical University, Xi'an, China; ^2^The Youth Innovation Team of Shaanxi Universities & School of Stomatology, Xi'an Medical University, Xi'an, China; ^3^Department of Implant Dentistry, College of Stomatology, Xi'an Jiaotong University, Xi'an, China; ^4^Lintong Rehabilitation and Convalescent Center, Joint Logistic Support Force, China People's Liberation Army, Xi'an, China; ^5^920th Hospital of Joint Logistics Support Force, Kunming, China

## Abstract

A functional vasculature for survival remains a challenge for tissue regeneration, which is indispensable for oxygen and nutrient supply. Utilizing mesenchymal stromal cells (MSCs) to alleviate tissue ischemia and repair dysfunctional or damaged endothelium is a promising strategy. Compared to other populations of MSCs, adipose-derived stromal cells (ASCs) possess a more significant proangiogenic potential and are abundantly available. Cell sheet technology has recently been widely utilized in bone engineering. Compared to conventional methods of seeding seed cell suspension onto biological scaffolds, cell sheet technology prevents cell loss and preserves the extracellular matrix (ECM). Nevertheless, the proangiogenic potential of ASC sheets remains unknown. In this study, rat ASC sheets were constructed, and their macro- and microstructures were examined. In addition, we investigated the effects of ASCs and ASC sheets on the biological properties and angiogenic capacity of endothelial cells (ECs). The results demonstrated that the ASC sheets gradually thickened as the number of cells and ECM increased over time and that the cells were in an active state of secretion. Similar to ASC-CM, the conditioned medium (CM) of ASC sheets could significantly enhance the proliferative capacity of ECs. ASC sheet-CM has significant advantages over ASC-CM in promoting the migration and angiogenesis of ECs, where the exosomes secreted by ASC sheets play an essential role. Therefore, using ASC sheets for therapeutic tissue and organ regeneration angiogenesis may be a valuable strategy.

## 1. Introduction

Tissue engineering technology is developing rapidly in regenerative medicine, providing a potential therapeutic approach for tissue and organ regeneration [1]. However, sufficient vascularization supplying oxygen and nutrients remains a challenge for tissue regeneration [2].

It has been reported that mesenchymal stromal cells (MSCs) express various cytokines and growth factors that promote tissue regeneration and vascularization [3–6]. In tissue regeneration, bone marrow-derived mesenchymal stromal cells (BMSCs) are among the most widely used cells. They are capable of self-regeneration and have multipotent properties [7]. However, its invasive isolation procedure and potential postoperative complications limit its clinical utility [8]. In recent years, interest in adipose-derived mesenchymal stromal cells (ASCs) has increased. ASCs are abundant and can be extracted by liposuction or lipotomy from adipose tissue all over the body [8]. Compared to BMSCs, ASCs have a greater proliferative capacity [9], enhanced colony frequency [10], improved genetic stability [11], and higher immunosuppression [12] and telomerase activity, as well as increased resistance to hypoxia-induced apoptosis and oxidative stress-induced senescence [13]. Besides, ASCs have a greater proangiogenic potential than BMSCs and other populations of MSCs [14–17]. As forming internal functional blood vessels is essential for the survival of regenerated tissue, the accessibility and angiogenesis advantages of ASCs make them a promising source of seed cells for tissue regeneration. Researchers reported that the conditioned medium (CM) of ASCs significantly enhanced the proliferative, migratory, and proangiogenic potentials of endothelial cells (ECs) [18–20]. However, little is known about the proangiogenic potential of ASC sheets at this time. In this study, we analyzed the effects of CM and exosomes on ECs to compare the proangiogenic capabilities of ASCs and ASC sheets.

## 2. Materials and Methods

### 2.1. Animals

All animal experimental procedures were conducted by the committee guidelines of the Laboratory Animal Care & Welfare Committee, School of Stomatology, Fourth Military Medical University, China. Eight-week-old male Sprague-Dawley rats (180-200 g) were purchased from the Chengdu Dossy Experimental Animals Company and used to isolate ASCs and animal experiments. The rats were housed in specific pathogen-free conditions with a temperature of 26°C and a humidity of 30-70%, and they were exposed to a standard light/dark cycle of 12 h of light followed by 12 h of dark throughout the study.

### 2.2. Cells and Cell Culture

#### 2.2.1. Rat Adipose-Derived Stromal Cells (ASCs)

ASCs were harvested from surgically extracted inguinal fat pads of rats by collagenase digestion as described previously [21] and cultured in T75 culture flasks with *α*-MEM (Gibco, USA) supplemented with 10% fetal bovine serum (Gibco, USA) and 1% penicillin/streptomycin (HyClone, USA). ASCs were incubated at 37°C in a humidified atmosphere of 5% CO_2_ and 95% air.

To evaluate the multilineage differentiation capacity of ASCs, osteogenic and adipogenic differentiation was carried out as described previously [21]. Briefly, the cells were seeded in six-well plates, and the culture medium was replaced with osteogenic medium or adipogenic medium when the cells reached 80% confluence. The osteoinductive medium was composed of 0.1 mM dexamethasone (Sigma, USA), 5 mM *β*-glycerophosphate (Sigma, USA), 50 *μ*g/mL L-ascorbic acid (Sigma, USA), in *α*-MEM (Gibco, USA) supplemented with 10% FBS (Gibco, USA) and 1% penicillin/streptomycin (HyClone, USA). The adipogenic medium was composed of *α*-MEM (Gibco, USA) containing 10% FBS (Sijiqing, China), 1% penicillin/streptomycin (HyClone, USA), 0.5 mM 3-isobutyl-1-methylxanthine (IBMX, France), 1 *μ*M dexamethasone (Sigma, USA), 0.1 mM indomethacin (Sigma, USA), and 10 *μ*g/mL insulin (Sigma, USA). The osteogenic or adipogenic induction medium was changed every 3 days. The calcium deposits yielded by the ASCs were visualized by Alizarin Red staining (Sigma, USA) after osteogenic induction for 28 days, while lipid droplets were revealed by Oil Red O staining (Sigma, USA) after adipogenic induction for 14 days.

Flow cytometry was used to determine the positive expression of ASC surface proteins CD29, CD34, CD45, and CD90 as described previously [21]. Briefly, some 1 × 10^6^ third passage ASCs were fixed with 4% paraformaldehyde for 15 min and then incubated with CD29, CD34 (eBioscience, USA), CD45, and CD90 (Abcam, USA) at room temperature for 1 h and then at 4°C in the dark. The labeled ASCs were assessed using a flow cytometer (Beckman Coulter, USA). The monoclonal antibodies CD29 and CD90 were used to identify the mesenchymal phenotype, and CD34 and CD45 were applied to exclude the hematopoietic and angiogenic lineages.

#### 2.2.2. Rat Endothelial Cells (ECs)

ECs were obtained from the same rats that were used for ASCs isolation. The thoracic aorta was obtained under sterile conditions. After being washed with phosphate-buffered saline (PBS) to remove the residual blood, the thoracic aorta with attached tissue was removed and was cut up into 2-4 mm pieces using a pair of microdissection scissors in a plate with endothelial cell growth medium (ECM) containing 1% endothelial cell growth supplement (ECGS), 5% fetal bovine serum, and 1% penicillin/streptomycin (ScienCell, USA). Then the aorta pieces were then immediately placed on the lumen side of a T25 culture flask using a pair of elbow tweezers and a dental probe. The culture flask was turned upside down and added with 2 mL ECM. After being incubated at 37°C in a humidified atmosphere of 5% CO_2_ and 95% air for 90-120 minutes, the culture flask was turned gently upside down again and kept in the cell incubator. ECM was added when necessary to keep the aortic segments wet. Once the confluency reached 80%, the aortic segments were gently removed. The cells were digested with 0.25% trypsin (HyClone, USA) and subcultured at a proportion of 1 : 2. Cells of the third passage were used for the subsequent experiments.

To examine the endothelial cell markers, immunofluorescence staining was performed. Briefly, endothelial cells were seeded in 24-well-chambered slides and cultured for 1-2 days. Then the cells were fixed with 4% paraformaldehyde at 4°C for 20 minutes, permeabilized with 0.1% Triton X-100 (Sigma, USA) at room temperature for 15 minutes, and blocked with goat serum (Solarbio, China) at room temperature for 30 minutes, and the cells were then incubated with the primary antibodies against CD31 (#ab24590, Abcam, USA) at a dilution of 1 : 100 or against von Willebrand factor (vWF) protein (#ab6994, Abcam, USA) in a dilution of 1 : 400 with the antibody dilution buffer overnight at 4°C. The secondary antibodies were applied at room temperature for 2 hours in the dark. Slides were mounted using a liquid mountant with DAPI (Invitrogen). The fluorescent images of the stained cells were captured under a fluorescence microscope.

### 2.3. Macromorphology and Microstructure of ASC Sheets

#### 2.3.1. ASC Sheets Preparation

The ASC sheets were fabricated as previously described [21]. Briefly, the third-generation ASCs were seeded at 1 × 10^6^ cells/well in 6-well plates. After reaching a confluency of about 90%, the basal medium was replaced by cell-sheet induction medium, which was composed of *α*-MEM (Gibco, USA), 10% bovine fetal serum (Gibco, USA), 1% penicillin/streptomycin (HyClone, USA), and 50 mg/mL vitamin C (Sigma, USA).

#### 2.3.2. Histological Observation of the Cell Sheets

On days 1, 4, 7, and 10 of cell sheet induction, respectively, the cell sheets were peeled off with cell scrapers or tweezers. The obtained cell sheets were fixed with 4% paraformaldehyde and underwent dehydration, embedding, and paraffin sectioning. And the HE staining, Sirius Red staining, and Masson staining were applied. The structure of the cell sheets was observed by microscope.

#### 2.3.3. Transmission Electron Microscopy (TEM) Observation

The 7-day ASC sheets were cut into 1.0 mm^3^ blocks and fixed with 2.5% glutaraldehyde overnight and 1% osmium acid for 2 hours. After being dehydrated and embedded, the sample pieces were cut into ultrathin slices. Then 3% uranyl acetate-citrate double staining was applied. The ultrastructure of the cell sheets was then observed by transmission electron microscopy (FEI Tecnai G2, USA).

### 2.4. The Effects of ASCs and ASC Sheets Conditioned Medium (CM) on the Biological Characteristics and Vascular Ability of ECs

#### 2.4.1. ASC-CM and ASC Sheet-CM Collection

The culture of the ASCs group and ASC sheet group were started with the third-generation ASCs seeded in 10 cm cell culture dishes. When the ASCs group reached a confluency of 90%, and the ASC sheet group had been induced for 7 days, the dishes were washed thoroughly with PBS and replenished with 10 mL serum-free *α*-MEM supplemented with 1% penicillin/streptomycin for 48 h before harvesting the medium. Collected medium samples were centrifuged at 1,000 rpm for 10 min and filtered through a 0.22 mm filter to remove cell debris and then were preserved at −80°C. As serum starvation may alter the expression patterns of secreted proteins, 5% fetal bovine serum was added to the media before being used in further experimentation. *α*-MEM supplemented with 5% fetal bovine serum and 1% penicillin/streptomycin was used as the control medium in this study. The 3 different groups were named (i) ASC-CM, (ii) ASC sheet-CM, and (iii) control medium.

#### 2.4.2. Endothelial Cell Proliferation Assay

*(1) Cell Counting Kit-8 (CCK-8) Assay*. To examine the effects of conditioned medium on ECs proliferation in vitro, the third-generation ECs were resuspended in ASC-CM, ASC sheet-CM, and control medium and seeded on 96-well plates at a concentration of 1 × 10^3^ cells/well. After 1-6 days, endothelial cell proliferation was detected, respectively, with the Cell Counting Kit-8 (CCK-8) cell viability kit according to the manufacturer's instructions. The absorbance at 450 nm was measured using a microplate reader.

*(2) 5-Ethynyl-20-deoxyuridine (EdU) Incorporation Assay*. The third-generation ECs were resuspended in ASC-CM, ASC sheet-CM, and control medium and seeded on 96-well plates at a concentration of 1 × 10^3^ cells/well. The newly synthesized DNA of the cells was assessed by the EdU incorporation assay using a BeyoClick™ EdU Cell Proliferation Kit with TMB (Beyotime, China), according to the manufacturer's instructions. Briefly, after being cultured for 72 h, the cells were incubated with 2 × EdU working liquid for two hours and washed with PBS 3 times. The cells were then fixed with 4% paraformaldehyde for 15 min, permeabilized with 0.3% Triton X-100 and 0.3% H_2_O_2,_ respectively, for 15 min and 20 min, and incubated in the dark successively with freshly prepared click additive solution and streptavidin-HRP working liquid, each for 30 min. Extensive washing with PBS was needed after each step mentioned above. After being incubated with TMB liquid for 30 min, the absorbance at 370 nm was detected using a microplate reader.

#### 2.4.3. The Effect of ASC-CM and ASC Sheet-CM on the Migration Ability of ECs

*(1) Scratch Plate Assay*. The third-generation ECs were resuspended in ASC-CM, ASC sheet-CM, and control medium and seeded on 6-well plates at a concentration of 5 × 10^5^ cells/well. When the cells reached a confluency of 80-90%, a sterile pipette tip of 200 *μ*L was used to draw a line on the internal surface of the well. Subsequently, the wells were washed twice with PBS and photographed under a microscope. After incubated in different culture media for 12 h or 24 h, each well was photographed again. The quantification was implemented by ImageJ software.

*(2) Transwell Assay*. This assay was performed on 24-well transwell plates, which were provided with a polycarbonate membrane with a porosity of 8 *μ*m. 500 *μ*L of ASC-CM, ASC sheet-CM, and control medium were added, respectively, to the lower chamber of the 24-well transwell plates. The third-generation ECs were resuspended in serum-free medium supplemented with 0.1% BSA and dispersed within the upper chamber of transwell dishes. After 48 hours of incubation at 37°C in a moistened atmosphere with 5% CO_2_, the surface of the membrane was rinsed with PBS and wiped with a cotton bud. The membrane was then fixed with 4% paraformaldehyde for 15 min and stained with DAPI for 5 min in the dark. The total number of cells that migrated was counted by the ImageJ software.

#### 2.4.4. The mRNA and Protein Expression of Vascular Endothelial Growth Factor (VEGF) of ECs

*(1) Real-Time Quantitative Polymerase Chain Reaction (qPCR)*. The third-generation ECs were seeded in 6-well plates at a concentration of 5 × 10^5^ cells/well and cultured with ASC-CM, ASC sheet-CM, and control medium, respectively. After 1, 4, and 7 days, total RNA was extracted from ECs with E.Z.N.A. Total RNA Kit (Omega Bio-Tek, USA) according to the manufacturer's protocol. After quantification by optical density measurement, the extracted RNA was converted to cDNA using a PrimeScript™ RT-PCR Kit (Takara, Japan). The RT-PCR was performed using TB Green Premix Ex Taq II(Tli RNaseH Plus) (Takara, Japan) in a quantitative PCR System (Bio-Rad, USA) with the primers for VEGF (forward, 5′-AGG AGT ACC CCG ATG AGA TA-3′; reverse, 5′-CTT CTA CTG CCC TCC TTG TA-3′) and GAPDH (forward, 5′- GGC ACA GTC AAG GCT GAG AAT G-3′; reverse, 5′- ATG GTG GTG AAG ACG CCA GTA -3′). GAPDH was monitored as a housekeeping gene. The results were evaluated by the CFX96TM RT-PCR System (Bio-Rad, USA). The 2^–*ΔΔ*Ct^ method was used to calculate the relative expression levels.

*(2) Western Blotting Analysis*. The third-generation ECs were seeded in 6-well plates at a concentration of 5 × 10^5^ cells/well and cultured with ASC-CM, ASC sheet-CM, and control medium for 7 days, respectively. Cells were harvested and lysed with RIPA buffer (Zhonghuihecai, China) supplemented with 1% phosphatase inhibitors. Protein concentrations were quantified by the BCA protein assay (Genshare, China). Proteins were separated by CFAS any KD PAGE Protein electrophoresis gel preparation kit (Zhonghuihecai, China) and transferred to the polyvinylidene fluoride (PVDF) membranes (Millipore, Germany). The membranes were blocked with 5% nonfat milk/Tris-buffered saline with tween (TBST) for 1 h and incubated with the primary antibodies, i.e., rat anti-VEGFA antibody (#32988, SAB, USA) and anti-GAPDH antibody (Ab-AF 7021, Affinity, China), at 4°C overnight. And the membranes were then incubated for 1 h with the secondary antibody of goat anti-Rabbit IgG (#18933-1-AP, SAB, USA). Immunoreactive proteins were visualized by chemiluminescence using electrochemiluminescence (ECL) agents (Millipore, Germany). The gray value of the protein bands was measured by the ImageJ software.

#### 2.4.5. Assessment of the Angiogenic Effect of ASC-CM and ASC Sheet-CM on ECs

*(1) Tube Formation Assay*. Twenty-four-well microplates were coated with 250 *μ*L per well of Matrigel (Corning, USA) and incubated for 1-2 h at 37°C to solidify. The third-generation ECs were resuspended in 500 *μ*L ASC-CM, ASC sheet-CM, and control medium and seeded on the plates at a concentration of 1.5 × 10^5^ cells/well and incubated at 37°C/5% CO_2_. After 6 h, the newly formed tubules were observed and recorded by a digital camera (Nikon, Japan) and evaluated using Image J software.

*(2) In Vivo Vasculogenic Assay*. To examine the in vivo vasculogenic potential of ASC-CM and ASC sheet-CM, a Matrigel plug assay was conducted. The third-generation ECs were seeded in 6-well plates at a concentration of 5 × 10^5^ cells/well and cultured with ASC-CM, ASC sheet-CM, and control medium, respectively. After 7 days, ECs from different groups were resuspended in PBS at a concentration of 6 × 10^6^/mL and mixed with Matrigel in a volume ratio of 1 : 4. PBS mixed with Matrigel at a volume ratio of 1 : 4 without cells was used for the blank control group. Animals were anesthetized by an intraperitoneal injection of 2% pentobarbital sodium solution (Sigma-Aldrich, USA) (0.25 mL/100 g body weight). Six rats were involved in the study. After being shaved and sterilized, their backs were crossed with a marker into 4 areas to be seeded with ECs from different groups, one for each of the 250 *μ*L mixtures from the blank group (left front), *α*-MEM group (right front), ASC-CM group (left back), and ASC sheet-CM group (right back), respectively, through subcutaneous injections. Three weeks later, the rats were sacrificed and Matrigel plugs were carefully removed, fixed with 4% formaldehyde, paraffin-embedded, and then sectioned for HE staining.

### 2.5. The Effects of Exosomes from ASCs and ASCs Sheet on the Biological Characteristics and Vascular Ability of ECs

#### 2.5.1. Exosomes Isolation and Identification

Preparation of exosome-free serum: centrifuge Gibco fetal bovine serum at 12000 g for 16 h, carefully aspirate the supernatant, filter it with a 0.22-*μ*m pore filter, and store it in a refrigerator at -20°C for later use.

For exosome isolation, the culture of both the ASC group and ASC sheet group was started with the third-generation ASCs seeded in 10 cm cell culture dishes. When the ASC group reached a confluency of 90% and the ASC sheet group had been induced for 7 days, the dishes were washed thoroughly with PBS and replenished with 10 mL *α*-MEM supplemented with 10% exosome-free serum and 1% penicillin/streptomycin for 48 h before harvesting the media.

Exosomes were isolated using the exosome precipitation kit (Applygen, China), according to the manufacturer's specifications. Culture media of the ASC group and ASC sheet group were centrifuged at 3000 g for 15 min and filtered through a 0.22*-μ*m filter (Millipore, USA) to remove dead cells and cellular debris. The supernatant (10 mL) was then mixed thoroughly with 2 mL of exosome extraction reagent and stored at 4°C overnight. The next day, the mixture was centrifuged at 1500 g for 30 min and 1500 g for another 5 min to obtain a pellet containing exosomes, which were then resuspended in 30-100 *μ*L PBS. The concentration of exosomes was measured using the BCA protein assay kit (Genshare, China) and then preserved at −80°C.

Exosome morphology was assessed via TEM (FEI Tecnai G2, USA) after being negatively stained with phosphotungstic acid. Exosomal surface markers, including CD81, CD63, Alix, and negative marker Calnexin, were detected by western blotting. A nanoparticle tracer analyzer (NTA, Malvern, UK) was used to measure the size distribution and density of exosomes from ASCs (ASC-exo) and ASC sheets (ASC sheet-exo).

#### 2.5.2. The Effects of ASC-Exo and ASC Sheet-Exo on the Migration Ability of ECs

Cell migration was measured using scratch plate assay and transwell assay following the previously established protocols in method 2.5. Briefly, ECs (5 × 10^5^/well) were seeded into 6-well plates, and scratches were made with a 200-*μ*L sterile tip. The medium was then replaced with 2 mL medium supplemented with 25 *μ*g/mL ASC exosomes or 25 *μ*g/mL ASC sheet exosomes per well. ECM medium without ECG was used in the control group. The three groups were called the ASC-exo group, the ASC sheet-exo group, and the control group. After being incubated in different culture media for 0 h, 12 h, and 24 h, each well was photographed under a microscope. The quantification was implemented by ImageJ software.

ECs were seeded into the upper transwell insert (pore size of 8 *μ*m) of a 24-well plate at a density of 1 × 10^4^ cells/200 *μ*L/well. 500 *μ*L ECM medium without ECG supplemented with 25 *μ*g/mL ASC-exo or 25 *μ*g/mL ASC sheet-exo were added to the lower chamber to incubate the cells for 48 h. ECM medium without ECG and exosomes was used in the control group. And then the cells in the transwell chamber were removed. After being stained with DAPI, the cells that had migrated into the lower transwell layer were counted by the ImageJ software.

#### 2.5.3. Assessment of the Angiogenic Effects of ASC-Exo and ASC Sheet-Exo on ECs

Angiogenic effects were measured using tube formation assay and in vivo vasculogenic assay following previously established protocols in method 2.7. Briefly, 250 *μ*L Matrigel was used to precoat each well of a 24-well plate and polymerized at 37°C. ECs were resuspended in an ECM medium without ECG and seeded into the plates at a concentration of 1.5 × 10^5^ cells/well. ASC or ASC sheet exosomes of 0, 10, 20, 40, 100, and 200 *μ*g/*μ*L were added to the medium, respectively. After being incubated at 37°C in an atmosphere of 5% CO2 for 6 h, the newly formed tubules were observed and recorded with a digital camera (Nikon, Japan) and evaluated using Image J software.

To examine the in vivo vasculogenic potential, a Matrigel plug assay was conducted. 200 *μ*g ASC exosomes, 200 *μ*g ASC sheet exosomes, or an equal volume of PBS were mixed with Matrigel on ice. Six rats were involved in this study. After being shaved and sterilized, their back was marked with an inverted T into 3 areas to be seeded with exosomes from different groups, one for each of the mixtures from the ASC-exo group (left front), the ASC sheet-exo group (right front), and the control group (back) through subcutaneous injections. Three weeks later, the rats were killed and Matrigel plugs were carefully removed, fixed in 4% formaldehyde, paraffin-embedded, and then sectioned for HE staining.

### 2.6. Statistical Analysis

All experiments were repeated at least 3 times independently. Data were expressed as means ± standard error of the means (SEM). Statistical significance was determined using either one-way or two-way ANOVA followed by Tukey's multiple comparison post hoc test in GraphPad Prism 8.0 (GraphPad Software). A *p* value of less than 0.05 was considered statistically significant.

## 3. Results

### 3.1. Characterization of ASCs and ECs

**Figure 1:**
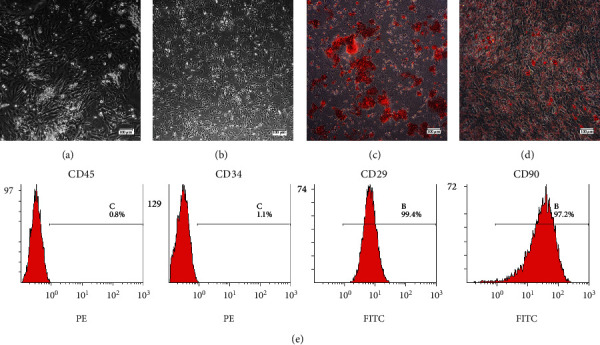
Characterization of ASCs. (a) Primary culture of ASCs. (b)Subculture of ASCs (P2). (c): Mineral nodes stained with Alizarin Red S. (d) Fat droplets stained with Oil Red O. (e) Flow cytometry analysis of ASC surface markers.

The primary ASCs presented as colonies with fibroblast-like spindle-shaped morphology (Figure 1(a)). The cell population appeared to be more homogeneous by the second passage (Figure 1(b)).

In osteogenic culture, calcium nodules were stained with Alizarin Red S (Figure 1(c)). In adipogenic culture, intercellular lipid vacuoles were stained with Oil Red O (Figure 1(d)). ASCs were negative for known hematopoietic or angiogenic markers CD45 and CD34, and positive for mesenchymal markers CD29 and CD90 (Figure 1(e)).

**Figure 2:**
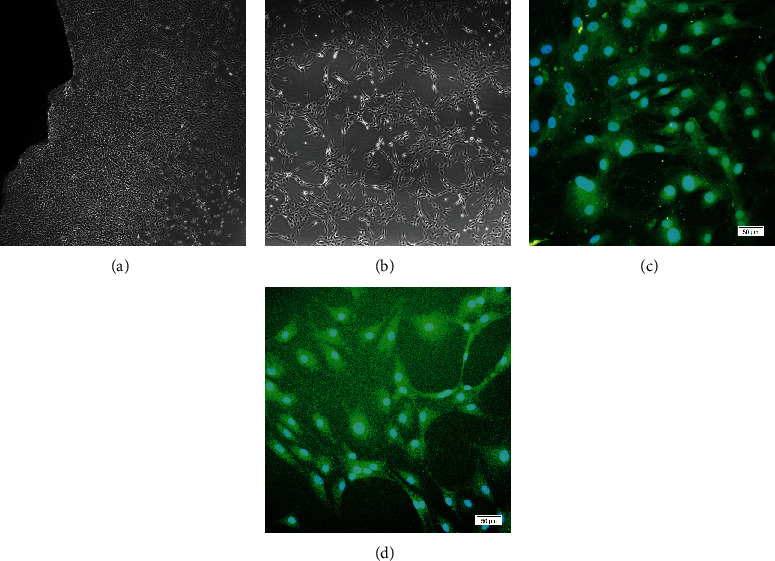
Characterization of ECs. (a) Primary culture of ECs. The black opaque area is the blood vessel fragments. (b) Subculture of ECs (P2). (c) ECs positively expressed vWF by immunofluorescence. (d) ECs positively expressed CD31 by immunofluorescence.

Endothelial cells formed typical cobblestone-like colonies after 7-10 days culture. The opaque black area is the blood vessel fragments, where the ECs crawled out (Figure 2(a)). Cells retained these phenotypes after the first passage. Most cultured ECs at confluence displayed the typical “cobblestone” appearance, whereas some were likely to form a tube-like structure (Figure 2(b)). The merged images under fluorescence microscopy observation showed that ECs positively expressed CD31 and vWF, and obvious vascular cavity-like structures were observed (Figures 2(c) and 2(d)).

### 3.2. Macromorphology and Microstructure of ASC Sheets

#### 3.2.1. The Morphology and Histological Staining of ASC Sheets

**Figure 3:**
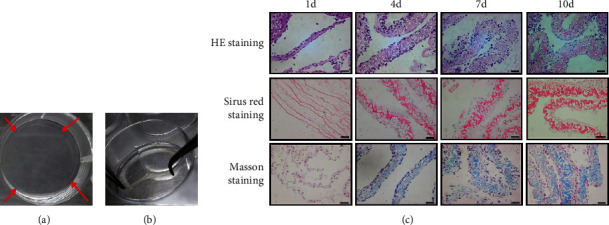
Morphological and histological staining of ASC sheets after the induction of vitamin C. (a) The edge of the ASC sheets began to curl slightly on day 7 of cell sheet induction (red arrows indicated the curly margins of ASC sheets). (b) ASC sheets could be easily detached by tweezers. (c) Histological staining of ASC sheets on days 1, 4, 7, and 10 of cell sheet induction, respectively. By HE staining, the cell nuclei were dyed blue and the collagen fibers pale pink. By Sirius Red staining, the collagen fibers were dyed red, and by Masson staining, the collagen fibers were stained blue. Scale bars: 20 *μ*m.

ASC sheets were gradually thickened with the induction of vitamin C. After 1 day of induction, the ASC sheets could hardly be detached intact and should be obtained only with a cell scraper. On day 3, ASC sheets began to take shape and could be easily detached by tweezers. On day 7, the edge of the cell sheets began to curl slightly (Figures 3(a) and 3(b)).

Representative photographs of HE staining, Sirius Red staining, and Masson staining are shown in Figure 3(c): The thickness of ASC sheets increased over time. The ASC sheets were about 5 *μ*m thick on the 1st day, 30-50 *μ*m thick on the 4th day, and 60-90 *μ*m thick on the 7th and 10th day, indicating an increasing thickness within 7 days to reach a maximum and stable one at last. Besides, both cells and collagen fibers of ASC sheets increased remarkably over time. ASC sheets presented a sandwich-like structure that consisted of a layer of cells, a layer of fibers, and another layer of cells in HE staining section. From day 4 to day 7, the middle fiber layer continued to thicken, while no obvious change in cell number was observed. And after 7 days of induction, the structure of ASC sheets remained stable. The same changes were observed in both Sirius Red staining and Masson staining.

#### 3.2.2. Transmission Electron Microscope (TEM) Observation

**Figure 4:**
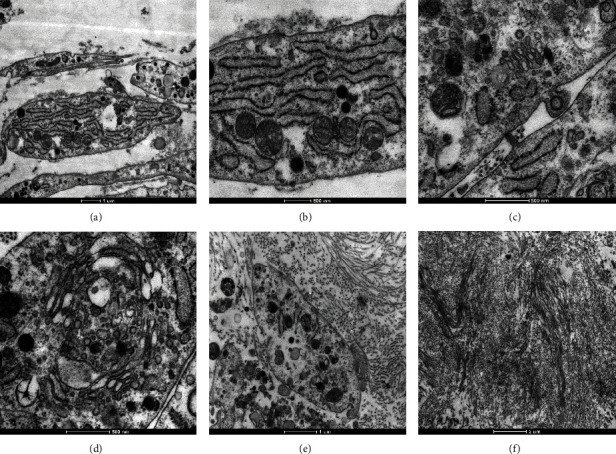
Transmission electron microscope (TEM) observation of ASC sheets on day 7. (a and b) Plenty of rough endoplasmic reticulum and ribosomes were observed in the cytoplasm of cells. (c and d) Abundant Golgi apparatus and exocytosis were observed. (e and f) A large number of collagen fibers could be seen in the ECM.

The ultrastructure of ASC sheets was observed by transmission electron microscope (TEM). Plenty of rough endoplasmic reticulum, ribosomes (Figures 4(a) and 4(b)), Golgi apparatus, and exocytosis (Figures 4(c) and 4(d)) were observed in the cytoplasm of ASCs, which demonstrated that the cells in the ASCs sheets were in an active state of secretion. Besides, a large number of collagen fibers could be seen in the ECM (Figures 4(e) and 4(f)).

### 3.3. ASC-CM and ASC Sheet-CM Accelerated the Proliferation of ECs

**Figure 5:**
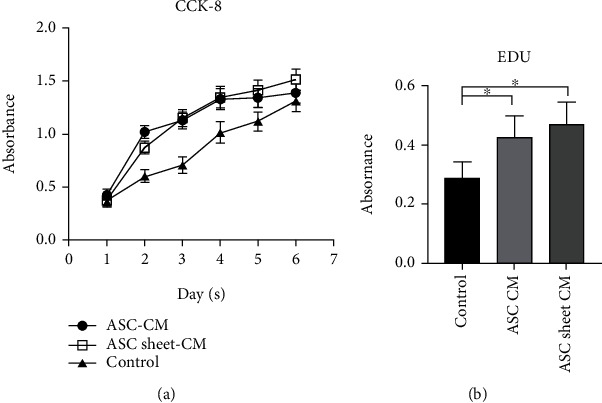
ASC-CM and ASC sheet-CM enhanced the proliferation of ECs. (a) The effects of ASC-CM and ASC sheet-CM on the proliferation of ECs were evaluated by Cell Counting Kit-8 (CCK-8) assay (*n* = 5). (b) The effects of ASC-CM and ASC sheet-CM on the proliferation of ECs were evaluated by Edu assay at 72 h. ^∗^*p* < 0.05.

We evaluated the effect of ASC-CM and ASC sheet-CM on the proliferation of ECs by CCK-8 and Edu assay. The CCK-8 assay revealed that the proliferation of ECs in the control group was significantly lower than that in the ASC-CM and ASC sheet-CM groups on days 2-6 (Figure 5(a)) (*p* < 0.05). The Edu assay displayed the same result at 72 h (Figure 5(b)). There is no significant difference between the ASC-CM group and the ASC sheet-CM group.

### 3.4. ASC Sheet-CM Showed a Stronger Ability to Promote the Migration of ECs Compared to ASC-CM

**Figure 6:**
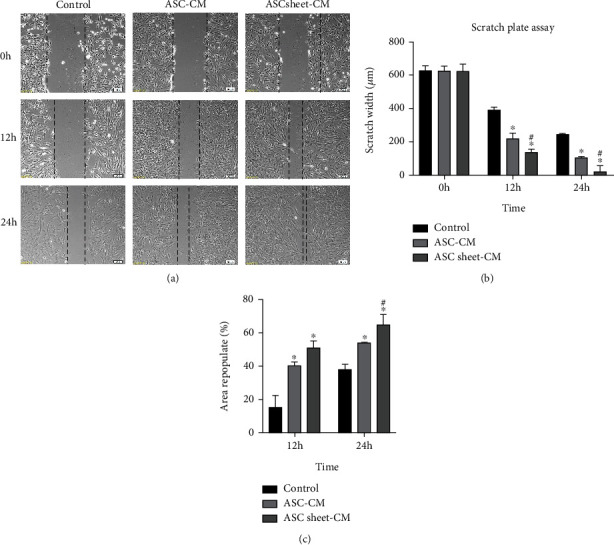
Effects of ASC-CM and ASC sheet-CM on the migration of ECs in scratch plate assay. (a) Representative images of the ECs scratch wound assay at 0 h, 12 h, and 24 h. Scale bars: 100 *μ*m. (b) The widths of the scratches at 0 h, 12 h, and 24 h. (c) The rate of area repopulation at 12 h and 24 h. ^∗^*p* < 0.05, versus the control group; #*p* < 0.05, versus the ASC-CM group.

The effects of ASC-CM and ASC sheet-CM on endothelial cell migration were analyzed by the scratch plate assay and the transwell assay. Both ASC-CM and ASC sheet-CM significantly promoted wound closure compared with the control medium, and the ASC sheet-CM group demonstrated the fastest healing rate among all groups (*p* < 0.05) (Figure 6).

**Figure 7:**
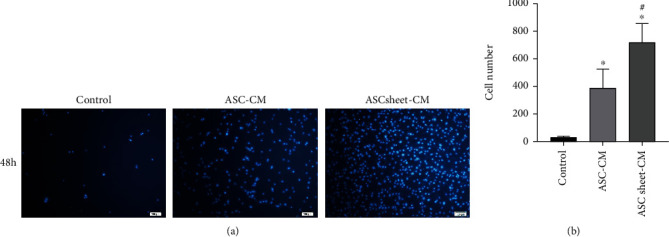
Effects of ASC-CM and ASC sheet-CM on the migration of ECs in the transwell assay. (a) Representative images of ECs that migrated through the membrane at 48 h. Scale bars: 100 *μ*m. (b) The number of migrated ECs at 48 h. ^∗^*p* < 0.05, versus the control group; #*p* < 0.05, versus the ASC-CM group.

After 48 hours of cell culture with each medium, the cells migrated through the membrane were counted. The numbers of migrated ECs were significantly higher in the ASC-CM group and the ASC sheet-CM group compared to that in the control group (*p* < 0.05), and the number of migrated ECs in the ASC sheet-CM group was the highest among all groups (*p* < 0.05) (Figure 7).

### 3.5. ASC Sheet-CM Upregulated the mRNA and Protein Expression of VEGF in ECs

**Figure 8:**
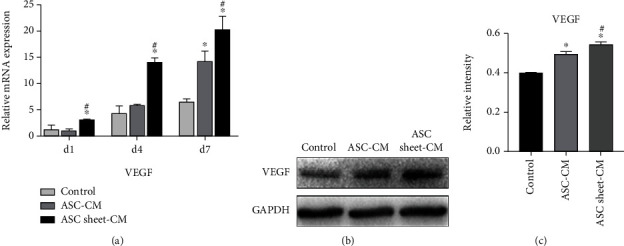
The effects of ASC-CM and ASC sheet-CM on the expression of VEGF in ECs (a) PCR analysis of the mRNA levels of VEGF in ECs on days 1, 4, and 7. (b) Western blotting analysis of protein levels of VEGF in ECs on day 7. (c) The gray values of the protein bands which were measured by the ImageJ software. ^∗^*p* < 0.05, versus the control group; #*p* < 0.05, versus the ASC-CM group.

The mRNA expression of VEGF in the ASC sheet-CM group was significantly higher than that in the other two groups on the first 1, 4, and 7 days (Figure 8(a)). The protein expression of VEGF was detected on the 7th day after ECs were treated with different media. The protein expression levels in both the ASC-CM group and the ASC sheet-CM group were higher than that in the control group (*p* < 0.05), and the ASC sheet-CM group had the highest expression level (*p* < 0.05) (Figures 8(b) and 8(c)).

### 3.6. ASC-CM and ASC Sheet-CM Promoted Angiogenesis Both In Vitro and In Vivo

**Figure 9:**
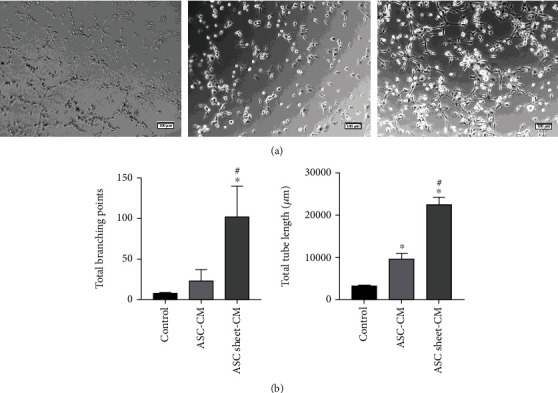
Angiogenic effects of ASC-CM and ASC sheet-CM in tube formation assay. (a) Representative images of ECs that formed tubes on Matrigel at 6 h. Scale bars: 100 *μ*m. (b) Graph showing the number of total branching points and total tube length. ^∗^*p* < 0.05, versus the control group; #*p* < 0.05, versus the ASC-CM group.

ECs can assemble spontaneously into tubules and form capillary-like structures when plated on Matrigel. Therefore, quantitative analysis of the tubules was used to quantify the ability of ECs to generate blood vessels in vitro. The results of the tube formation assay showed that the number of branch points and the total tube length in the ASC-CM and the ASC sheet-CM group were significantly higher than those of the control group (*p* < 0.05), and the ASC sheet-CM group displayed a significantly the longest tube length and most branch points (*p* < 0.05) (Figure 9).

**Figure 10:**
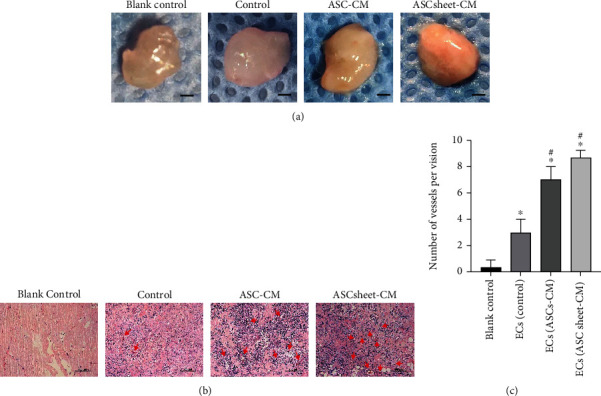
Angiogenic effects of ASC-CM and ASC sheet-CM in Matrigel plug assay in vivo. (a) Macroscopic view of representative explants at 3 weeks. Matrigel plug containing ECs treated with different media was subcutaneously injected into the dorsum of rats. The explants were excavated after 3 weeks. The group of Matrigel plugs without ECs was used as the control. (b) New vascular formation of the explant sections was detected by H&E staining; red arrows indicate blood vessels. Scale bars: 100 *μ*m. (c) Statistical analysis of microvessel numbers in Matrigel explants. ^∗^*p* < 0.05, versus the blank control group; #*p* < 0.05, versus the ECs group (control group).

To further assess the proangiogenic capacities of ASC-CM and ASC sheet-CM in vivo, Matrigel plug containing ECs was injected into rats' the dorsum subcutaneously. Three weeks following the injection, the Matrigel plugs were stripped off. As shown in Figure 10(a), the red fields and shades were different among different groups. The explants of the blank control group were translucent. The explants of the ASC-CM group and the ASC sheet-CM group were red, and the explants of the ASC sheet-CM group showed notably reddest areas. Subsequently, to confirm the blood vessel origin, histologic analysis was performed using HE staining under a light microscope. As shown in Figures 10(b) and 10(c), the results of histology revealed the differences in the vessel numbers among different groups. ECs in the control group only formed very few vessels. On the other hand, ASC-CM and ASC sheet-CM promoted significantly increased vessel numbers (*p* < 0.05). However, no statistical difference was observed in the ASC sheet-CM group compared with the ASC-CM group (*p* > 0.05). These results indicated that ASC-CM and ASC sheet-CM promoted the microvessel formation of ECs and accelerated tissue vascularization in vivo.

### 3.7. Characterization of ASC-Exo and ASC Sheet-Exo

**Figure 11:**
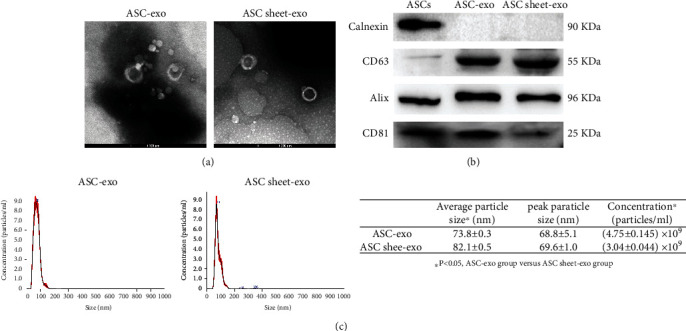
Characterization of ASC-exo and ASC sheet-exo. (a) Representative TEM images of ASC-exo and ASC sheet-exo. (b) Western blotting analysis of exosomal marker proteins (CD63, Alix, and CD81), and the negative marker (Calnexin) of ASC-exo and ASC sheet-exo. (c) Particle size distribution of ASC-exo and ASC sheet-exo measured by NTA.

Representative TEM images of ASC-exo and ASC sheet-exo were shown in Figure 11(a). The exosomes exhibited bilayer membranes and cup- or round-shaped structures. Western blot analysis revealed that both ASC-exo and ASC sheet-exo expressed exosome-specific markers CD63, Alix, and CD81, but not Calnexin, an integral protein of the endoplasmic reticulum that is not expressed in exosomes (Figure 11(b)). Nanoparticle tracking analysis (NTA) showed that the average particle size of ASC sheet-exo (82.1 ± 0.5 nm) was bigger than that of ASC-exo (73.8 ± 0.3 nm) (*p* < 0.05). However, the concentration of ASC-exo ((4.75 ± 0.145) × 10^9^ particles/mL) was greater than that of ASC sheet-exo ((3.04 ± 0.044) × 10^9^ particles/mL) (*p* < 0.05). The modal peak of ASC-exo (68.8 ± 5.1 nm) and ASC sheet-exo (69.6 ± 1.0 nm) had no significant difference (*p* > 0.05) (Figure 11(c)).

### 3.8. ASC Sheet-Exo Showed a Stronger Ability to Promote the Migration of ECs Compared to ASC-Exo

**Figure 12:**
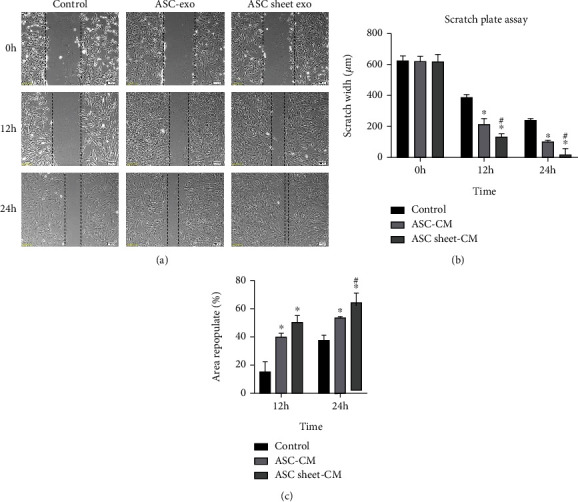
Effects of ASC-exo and ASC sheet-exo on the migration of ECs in scratch plate assay. (a) Representative images of ECs scratch wound assay at 0 h, 24 h, and 48 h. Scale bars: 100 *μ*m. (b) The widths of the scratches at 0 h, 24 h, and 48 h. (c) The rates of area repopulation at 24 h and 48 h. ^∗^*p* < 0.05, versus the control group; #*p* < 0.05, versus the ASC-exo group.

The effects of ASC-exo and ASC sheet-exo on endothelial cell migration were analyzed by the scratch plate assay and the transwell assay. Both ASC-exo and ASC sheet-exo significantly increased wound closure compared with the control medium, and ASC sheet-exo demonstrated the fastest healing rate among all groups (*p* < 0.05) (Figure 12).

**Figure 13:**
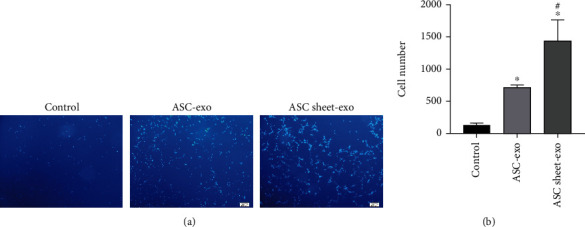
Effects of ASC-exo and ASC sheet-exo on the migration of ECs in the transwell assay. (a) Representative images of ECs that had migrated through the membrane at 48 h. Scale bars: 100 *μ*m. (b) The number of migrated ECs at 48 h. ^∗^*p* < 0.05, versus the control group; ^#^*p* < 0.05, versus the ASC-exo group.

After 48 hours' exposure to ASC-exo and ASC sheet-exo of the same concentration, the cells that had migrated through the membrane were counted. The number of migrated ECs was significantly higher in the ASC-exo and the ASC sheet-exo group compared to that in the control group (*p* < 0.05), and the number of migrated ECs in the ASC sheet-exo group was the highest among all groups (*p* < 0.05) (Figure 13).

### 3.9. ASC Sheet-Exo Displayed Higher Proangiogenic Potential Compared to ASC-Exo

**Figure 14:**
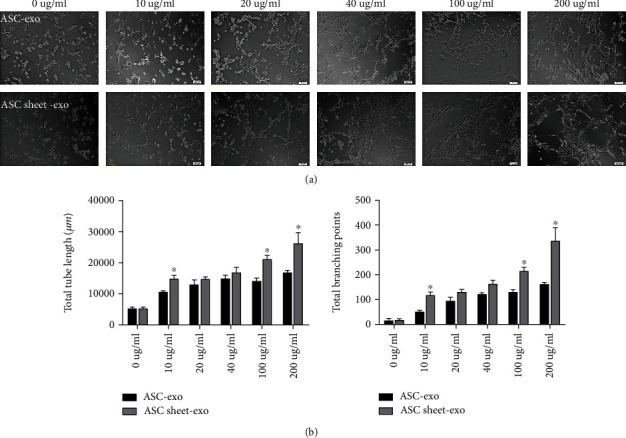
Angiogenic effects of ASC-exo and ASC sheet-exo in tube formation assay. (a) Representative images of ECs that had newly formed tubes on Matrigel at 6 h. Scale bars: 100 *μ*m. (b) Graph showing the number of total branching points and total tube length. ^∗^*p* < 0.05, versus the control group; ^#^*p* < 0.05, versus the ASC-exo group.

The total lumen length and the number of branch points in the ASC sheet-exo group were significantly higher than those in the ASC-exo group when the exosomes in both groups were provided at the same concentration of 10, 100, and 200 g/L (*p* < 0.05). The total lumen length and the number of branch points in the ASC-exo group and the ASC sheet-exo group with different exosome concentrations were significantly higher than those in the group without any exosomes (*p* < 0.05). With the increase in exosome concentration, the tube-forming effects of both the ASC-exo group and ASC sheet-exo group were enhanced (Figure 14).

**Figure 15:**
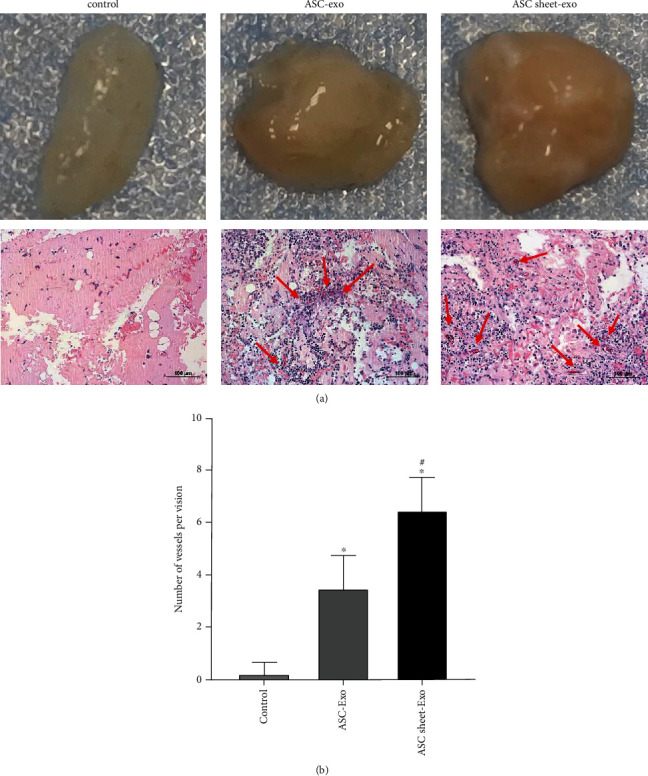
Angiogenic effects of ASC-exo and ASC sheet-exo in Matrigel plug assay in vivo. (a) Macroscopic view of representative explants and microscopic examination of H&E-stained paraffin sections at 3 weeks. Scale bars: 100 *μ*m. (b) Statistical analysis of microvessel number in Matrigel explants. ^∗^*p* < 0.05, versus the blank control group; ^#^*p* < 0.05, versus the control group.

To further assess the proangiogenic capacities of ASC-exo and ASC sheet-exo in vivo, Matrigel plugs containing 200 *μ*g exosomes were injected into rats' dorsum subcutaneously. Three weeks after the injection, the Matrigel plug was stripped off. As shown in Figure 15(a), the explants from the ASC-exo group and the ASC sheet-exo group were red, and the explants from the ASC sheet-exo group were reddest. The results of histology revealed that ASC-exo and ASC sheet-ex increased vessel numbers significantly (*p* < 0.05), and ASC sheet-exo accelerated tissue vascularization most efficiently (Figure 15(b)).

## 4. Discussion

Cellular therapy is a promising approach in the field of tissue regeneration. However, treatment success depends not only on the differentiation potential of MSCs but also on the effectiveness of angiogenesis [22]. MSCs can affect cell survival and angiogenesis by secreting vascular-related factors and cytoprotective factors, which are essential to functional tissue regeneration [23, 24]. Unfortunately, traditional tissue engineering, in which seed cells are planted on biological scaffolds, has several drawbacks. On the one hand, protease digestion impairs the activity and function of cells [25]. On the other hand, cells are lost during the process of inoculating cell suspension onto biological scaffold materials [26]. Fortunately, cell sheet technology efficiently resolved these issues. It can not only preserve the surface proteins of the MSCs but also reduce cell loss and retain a substantial amount of extracellular matrix (ECM), thereby improving the microenvironment of the MSCs [27, 28].

MSC-CM contains active proteins, exosomes, and genetic materials [23]. Numerous studies [14–17, 29, 30] have demonstrated that the CM of ASCs is more effective than other sources of MSCs at promoting angiogenesis, indicating that ASCs are an ideal cell source for treating ischemic diseases and tissue-engineered regeneration. However, little is known about the proangiogenic potential of ASC sheets at this time. Therefore, we constructed rat ASC sheets, examined their macro- and microstructures, and compared the angiogenic potential of ASC sheets to that of ASCs.

When sheet-forming ASCs were seeded in large numbers (1 × 10^6^ cells per well in 6-well plates), cell sheets were formed only three days after induction and gradually thickened to reach 30-50 *μ*m thick on the fourth day and 60-90 *μ*m on the seventh and tenth days. Unlike previous studies [31–33] in which ASCs were cultured at a much lower density, ASC sheets of only 32.6 ± 5.0 *μ*m and 52.2 ± 6.8 *μ*m thick were harvested, respectively, after one- and three-week induction of vitamin C [33]. We, therefore, hypothesize that a culture with a high density of ASCs favors the fabrication of cell sheets, with a faster induction rate and superior results. However, additional research is required to confirm this hypothesis.

Histological staining revealed that the number of ASCs within the cell sheets increased significantly during the first three days of induction and remained stable, possibly due to contact inhibition [34]. In contrast, the collagen fibers continued to increase until the seventh day, when they reached their maximum. Consistent with a previous report [33], it can be a good signal for the final maturation of ASC sheets on the seventh day. It implies a minimal requirement for 7-day induction to achieve the best sheet-forming effect. Moreover, it suggests a potentially reliable preparation time for clinical application with 1 × 10^6^ cells per well (6-well plate). Therefore, the ASC sheets of 7-day induction were utilized in all subsequent experiments.

Additionally, TEM observation was performed further to investigate the structure and function of ASC sheets. ASCs in the sheets were functionally active and vigorously secretory, with abundant rough endoplasmic reticulum, ribosomes, Golgi apparatus, and exocytosis vesicles in the cytoplasm. Consistent with the thriving state of ASCs, significant collagen fibers were deposited in the ECM.

To examine the effects of ASCs and ASC sheets on the biological properties and proangiogenic potential of ECs, we compared ASC sheet-CM and ASC-CM. The results demonstrated that ASC sheet-CM could promote EC proliferation similarly to ASC-CM, and it has clear advantages in enhancing the migratory and proangiogenic potential of ECs compared to ASC-CM.

It may be because sheet-forming induction significantly increased cell density, and ASC sheets may contain more angiogenic-related factors. Sukho et al. [35] discovered that the CM of high-density ASCs contained more angiogenic factors (VEGF and fibroblast growth factor (FGF)) and fewer proinflammatory genes (tumor necrosis factor alpha (TNF*α*) and prostaglandin synthase 2 (PTGS2)) compared to the CM of low-density ASCs. Bhang et al. demonstrated that the concentrations of angiogenic and antiapoptotic factors (VEGF, FGF, HGF, and chemokine (C-X-C motif) ligand 12 (CXCL12)) in the CM of 3D spheroid ASCs were tens of times greater than those in ASC-CM produced by conventional monolayer culture. The density of ASCs cultured in a 3D sphere culture system was approximately four times that of conventional monolayer culture [36].

The ECM may also play a significant role. Deng et al. cultured ASCs, adipose-derived stromal vascular component (SVF), and ECM/SVF with the same number of cells and collected the supernatants to prepare CM. ECM/SVF was found to secrete the highest concentrations of growth factors (i.e., VEGF, FGF, HGF, and transforming growth factor-*β* (TGF-*β*)) and significantly improve wound healing effect [37]. ASC sheets are richer in ECM than ASCs without induction, providing physical support for cells and a favorable microenvironment for cell function. It may be another important reason why ASC sheet-CM promotes angiogenesis more effectively.

Exosomes derived from ASCs transport an abundance of proteins, lipids, ribonucleic acids, and genomic and mitochondrial DNA and play a crucial role in intercellular communication [38]. Numerous studies have demonstrated that exosomes derived from ASCs can promote the proliferation and migration of ECs, induce the formation of vascular-like structures in vitro, and promote angiogenesis in animals [39–41]. In addition, they have exhibited good efficacy in flap repairment [40] and the treatment of kidney ischemia-reperfusion injury [42], myocardial infarction [43], cerebral ischemia [44], lung injury [41, 45], and diabetic limb ischemia [39]. However, reports showed that different cell sources [40] and treatment conditions [46–48] could affect the quantity and composition of exosomes. Therefore, we investigated the differences between ASC-exo and ASC sheet-exo in terms of particle size, concentration, and effects on the migration and angiogenesis of ECs. The concentration of ASC sheet-exo was lower than that of ASC-exo, but the average particle size of ASC sheet-exo was greater. It indicated that the bioactive components of ASC sheet-exo and ASC-exo were distinct, which may account for their distinct abilities (ASC sheet-exo promotes EC migration and angiogenesis better than ASC-exo).

This study has several limitations worth mentioning. First, both in vitro and in vivo experiments have confirmed that ASC sheets have greater proangiogenic potential. However, this study only examined the phenomenon and could not determine whether it was due to cell number, cytokines, or the extracellular matrix. In addition, additional experiments are required to confirm the content and function differences between ASC sheet-exo and ASC-exo. Finally, this study only addressed the proangiogenic potential. It did not examine other fields, such as wound healing indicators, chondrogenesis, or osteogenic ability.

## 5. Conclusion

In terms of promoting the migration and angiogenesis of ECs, the present study demonstrated that ASC sheet-CM has significant advantages over ASC sheet-CM in which exosomes secreted by ASC sheets play a significant role. Therefore, using ASC sheets for therapeutic tissue and organ regeneration angiogenesis may be a helpful strategy.

## Data Availability

The data used to support the findings of this study are available from the corresponding author upon reasonable request.

## References

[1] Hassanzadeh P., Atyabi F., Dinarvand R. (2018). Tissue engineering: still facing a long way ahead. *Journal of Controlled Release: Official Journal of the Controlled Release Society*.

[2] Li Y., Fraser D., Mereness J. (2022). Tissue engineered neurovascularization strategies for craniofacial tissue regeneration. *ACS Applied Bio Materials*.

[3] Schott N. G., Friend N. E., Stegemann J. P. (2021). Coupling osteogenesis and vasculogenesis in engineered orthopedic tissues. *Tissue Engineering. Part B, Reviews*.

[4] Sun J., Shen H., Shao L. (2020). HIF-1*α* overexpression in mesenchymal stem cell-derived exosomes mediates cardioprotection in myocardial infarction by enhanced angiogenesis. *Stem Cell Research & Therapy*.

[5] Diomede F., Marconi G. D., Fonticoli L. (2020). Functional relationship between osteogenesis and angiogenesis in tissue regeneration. *International Journal of Molecular Sciences*.

[6] Melchiorri A. J., Nguyen B. N., Fisher J. P. (2014). Mesenchymal stem cells: roles and relationships in vascularization. *International Journal of Molecular Sciences*.

[7] Leone A. M., Valgimigli M., Giannico M. B. (2009). From bone marrow to the arterial wall: the ongoing tale of endothelial progenitor cells. *European Heart Journal*.

[8] Panetta N. J., Gupta D. M., Longaker M. T. (2010). Bone regeneration and repair. *Current Stem Cell Research & Therapy*.

[9] Dmitrieva R. I., Minullina I. R., Bilibina A. A., Tarasova O. V., Anisimov S. V., Zaritskey A. Y. (2012). Bone marrow- and subcutaneous adipose tissue-derived mesenchymal stem cells: differences and similarities. *Text*.

[10] Kern S., Eichler H., Stoeve J., Kluter H., Bieback K. (2006). Comparative analysis of mesenchymal stem cells from bone marrow, umbilical cord blood, or adipose tissue. *Stem cells (Dayton, Ohio)*.

[11] Meza-Zepeda L. A., Noer A., Dahl J. A., Micci F., Myklebost O., Collas P. (2008). High-resolution analysis of genetic stability of human adipose tissue stem cells cultured to senescence. *Journal of Cellular and Molecular Medicine*.

[12] Zhou W., Lin J., Zhao K. (2019). Single-cell profiles and clinically useful properties of human mesenchymal stem cells of adipose and bone marrow origin. *The American Journal of Sports Medicine*.

[13] El-Badawy A., Amer M., Abdelbaset R. (2016). Adipose stem cells display higher regenerative capacities and more adaptable electro-kinetic properties compared to bone marrow-derived mesenchymal stromal cells. *Scientific Reports*.

[14] Jervis M., Huaman O., Cahuascanco B. (2019). Comparative analysis of in vitro proliferative, migratory and pro-angiogenic potentials of bovine fetal mesenchymal stem cells derived from bone marrow and adipose tissue. *Veterinary Research Communications*.

[15] Lu H., Wang F., Mei H., Wang S., Cheng L. (2018). Human adipose mesenchymal stem cells show more efficient angiogenesis promotion on endothelial colony-forming cells than umbilical cord and endometrium. *Stem Cells*.

[16] Hsiao S. T., Asgari A., Lokmic Z. (2012). Comparative analysis of paracrine factor expression in human adult mesenchymal stem cells derived from bone marrow, adipose, and dermal tissue. *Stem Cells and Development*.

[17] Kim Y., Kim H., Cho H., Bae Y., Suh K., Jung J. (2007). Direct comparison of human mesenchymal stem cells derived from adipose tissues and bone marrow in mediating neovascularization in response to vascular ischemia. *Cellular Physiology and Biochemistry : International Journal of Experimental Cellular Physiology, Biochemistry, and Pharmacology*.

[18] Delfi I., Wood C. R., Johnson L. D. V. (2020). An in vitro comparison of the neurotrophic and angiogenic activity of human and canine adipose-derived mesenchymal stem cells (MSCs): translating MSC-based therapies for spinal cord Injury. *Injury*.

[19] Jing X., Yin W., Tian H. (2018). Icariin doped bioactive glasses seeded with rat adipose-derived stem cells to promote bone repair _via_ enhanced osteogenic and angiogenic activities. *Life Sciences*.

[20] Zhong Z., Gu H., Peng J. (2016). GDNF secreted from adipose-derived stem cells stimulates VEGF-independent angiogenesis. *Oncotarget*.

[21] Xu X., Fang K., Wang L., Liu X., Zhou Y., Song Y. (2019). Local application of semaphorin 3A combined with adipose-derived stem cell sheet and anorganic bovine bone granules enhances bone regeneration in type 2 diabetes mellitus rats. *Stem Cells International*.

[22] Reddy L. V. K., Murugan D., Mullick M., Begum Moghal E. T., Sen D. (2020). Recent approaches for angiogenesis in search of successful tissue engineering and regeneration. *Current Stem Cell Research & Therapy*.

[23] Maacha S., Sidahmed H. (2020). Paracrine mechanisms of mesenchymal stromal cells in angiogenesis. *Stem Cells International*.

[24] Liu Y., Zhuang X., Yu S. (2021). Exosomes derived from stem cells from apical papilla promote craniofacial soft tissue regeneration by enhancing Cdc42-mediated vascularization. *Stem Cell Research & Therapy*.

[25] Li M., Ma J., Gao Y., Yang L. (2019). Cell sheet technology: a promising strategy in regenerative medicine. *Cytotherapy*.

[26] Yang J., Yamato M., Shimizu T. (2007). Reconstruction of functional tissues with cell sheet engineering. *Biomaterials*.

[27] Yang J., Yamato M., Nishida K. (2006). Cell delivery in regenerative medicine: the cell sheet engineering approach. *Journal of Controlled Release: Official Journal of the Controlled Release Society*.

[28] Elloumi-Hannachi I., Yamato M., Okano T. (2010). Cell sheet engineering: a unique nanotechnology for scaffold-free tissue reconstruction with clinical applications in regenerative medicine. *Journal of Internal Medicine*.

[29] Ikegame Y., Yamashita K., Hayashi S. (2011). Comparison of mesenchymal stem cells from adipose tissue and bone marrow for ischemic stroke therapy. *Cytotherapy*.

[30] Blasi A., Martino C., Balducci L. (2011). Dermal fibroblasts display similar phenotypic and differentiation capacity to fat-derived mesenchymal stem cells, but differ in anti-inflammatory and angiogenic potential. *Vascular cell*.

[31] Wei F., Qu C., Song T. (2012). Vitamin C treatment promotes mesenchymal stem cell sheet formation and tissue regeneration by elevating telomerase activity. *Journal of Cellular Physiology*.

[32] Yu J., Tu Y. K., Tang Y. B., Cheng N. C. (2014). Stemness and transdifferentiation of adipose-derived stem cells using L-ascorbic acid 2-phosphate-induced cell sheet formation. *Biomaterials*.

[33] Neo P. Y., See E. Y., Toh S. L., Goh J. C. (2016). Temporal profiling of the growth and multi-lineage potentiality of adipose tissue-derived mesenchymal stem cells cell-sheets. *Journal of Tissue Engineering and Regenerative Medicine*.

[34] McClatchey A. I., Yap A. S. (2012). Contact inhibition (of proliferation) redux. *Current Opinion in Cell Biology*.

[35] Sukho P., Kirpensteijn J., Hesselink J. W., van Osch G. J., Verseijden F., Bastiaansen-Jenniskens Y. M. (2017). Effect of cell seeding density and inflammatory cytokines on adipose tissue-derived stem cells: an in vitro study. *Stem Cell Reviews and Reports*.

[36] Bhang S. H., Lee S., Shin J. Y., Lee T. J., Jang H. K., Kim B. S. (2014). Efficacious and clinically relevant conditioned medium of human adipose-derived stem cells for therapeutic angiogenesis. *Molecular therapy : the journal of the American Society of Gene Therapy*.

[37] Deng C., He Y., Feng J. (2017). Extracellular matrix/stromal vascular fraction gel conditioned medium accelerates wound healing in a murine model. *Oxidative Medicine and Cellular Longevity*.

[38] Trzyna A., Banaś-Ząbczyk A. (2021). Adipose-derived stem cells secretome and its potential application in "stem cell-free therapy". *Biomolecules*.

[39] Zhang X., Jiang Y., Huang Q. (2021). Exosomes derived from adipose-derived stem cells overexpressing glyoxalase-1 protect endothelial cells and enhance angiogenesis in type 2 diabetic mice with limb ischemia. *Stem Cell Research & Therapy*.

[40] Xiong J., Liu Z., Wu M., Sun M., Xia Y., Wang Y. (2020). Comparison of proangiogenic effects of adipose-derived stem cells and foreskin fibroblast exosomes on artificial dermis prefabricated flaps. *Stem Cells International*.

[41] Mizuta Y., Akahoshi T., Guo J. (2020). Exosomes from adipose tissue-derived mesenchymal stem cells ameliorate histone-induced acute lung injury by activating the PI3K/Akt pathway in endothelial cells. *Stem Cell Research & Therapy*.

[42] Lin K. C., Yip H. K., Shao P. L. (2016). Combination of adipose-derived mesenchymal stem cells (ADMSC) and ADMSC- derived exosomes for protecting kidney from acute ischemia-reperfusion injury. *International Journal of Cardiology*.

[43] Waters R., Alam P., Pacelli S., Chakravarti A. R., Ahmed R. P. H., Paul A. (2018). Stem cell-inspired secretome-rich injectable hydrogel to repair injured cardiac tissue. *Acta Biomaterialia*.

[44] Yang Y., Cai Y., Zhang Y., Liu J., Xu Z. (2018). Exosomes secreted by adipose-derived stem cells contribute to angiogenesis of brain microvascular endothelial cells following oxygen-glucose deprivation in vitro through microRNA-181b/TRPM7 axis. *Journal of molecular neuroscience : MN*.

[45] Zhang C., Wang P., Mohammed A., Zhou Z. (2019). Function of adipose-derived mesenchymal stem cells in monocrotaline-induced pulmonary arterial hypertension through miR-191 via regulation of BMPR2. *BioMed Research International*.

[46] Bai Y., Han Y. D., Yan X. L. (2018). Adipose mesenchymal stem cell-derived exosomes stimulated by hydrogen peroxide enhanced skin flap recovery in ischemia-reperfusion injury. *Stem Cells International*.

[47] Kang T., Jones T. M., Naddell C. (2016). Adipose-derived stem cells induce angiogenesis via microvesicle transport of miRNA-31. *Stem Cells Translational Medicine*.

[48] Lopatina T., Bruno S., Tetta C., Kalinina N., Porta M., Camussi G. (2014). Platelet-derived growth factor regulates the secretion of extracellular vesicles by adipose mesenchymal stem cells and enhances their angiogenic potential. *Journal of molecular neuroscience : MN*.

